# Anaerobic utilization of pectinous substrates at extremely haloalkaline conditions by *Natranaerovirga pectinivora* gen. nov., sp. nov., and *Natranaerovirga hydrolytica* sp. nov., isolated from hypersaline soda lakes

**DOI:** 10.1007/s00792-012-0431-6

**Published:** 2012-02-01

**Authors:** D. Y. Sorokin, T. P. Tourova, A. N. Panteleeva, E. N. Kaparullina, G. Muyzer

**Affiliations:** 1 Winogradsky Institute of Microbiology, Russian Academy of Sciences, Prospect 60-let Octyabrya 7/2, 117811 Moscow, Russia; 2 Environmental Biotechnology Group, Department of Biotechnology, Delft University of Technology, Delft, The Netherlands; 3 Bioengineering Centre, Russian Academy of Sciences, Prospect 60-let Octyabrya 7/1, 117811 Moscow, Russia; 4 Institute of Physiology and Biochemistry of Microorganisms, Russian Academy of Sciences, Puschino, Russia; 5 Present Address: Department of Aquatic Microbiology, Institute for Biodiversity and Ecosystem Dynamics, University of Amsterdam, Amsterdam, The Netherlands

**Keywords:** Pectin, Pectinolytic, Haloalkaliphilic, Soda lakes

## Abstract

Anaerobic enrichments at pH 10, with pectin and polygalacturonates as substrates and inoculated with samples of sediments of hypersaline soda lakes from the Kulunda Steppe (Altai, Russia) demonstrated the potential for microbial pectin degradation up to soda-saturating conditions. The enrichments resulted in the isolation of six strains of obligately anaerobic fermentative bacteria, which represented a novel deep lineage within the order *Clostridiales* loosely associated with the family *Lachnospiraceae*. The isolates were rod-shaped and formed terminal round endospores. One of the striking features of the novel group is a very narrow substrate spectrum for growth, restricted to galacturonic acid and its polymers (e.g. pectin). Acetate and formate were the final fermentation products. Growth was possible in a pH range from 8 to 10.5, with an optimum at pH 9.5–10, and in a salinity range from 0.2 to 3.5 M Na^+^. On the basis of unique phenotypic properties and distinct phylogeny, the pectinolytic isolates are proposed to be assigned to a new genus *Natranaerovirga* with two species *N. hydrolytica* (APP2^T^=DSM24176^T^=UNIQEM U806^T^) and *N. pectinivora* (AP3^T^=DSM24629^T^=UNIQEM U805^T^).

**Electronic supplementary material:**

The online version of this article (doi:10.1007/s00792-012-0431-6) contains supplementary material, which is available to authorized users.

## Introduction

Pectin, a natural polymer of galacturonic acid, is an important component of plant biomass as a part of hemicelluloses. It is degraded by pectinolytic microorganisms producing an extracellular enzymatic complex consisting of exo- and endopectinases, pectate lyase and methylesterase (Jayani et al. 2005). The pectin hydrolysis is an important process, both for natural habitats and for industrial processing of food and textile (Kashyap et al. 2001; Sarethy et al. 2011). The first discovered pectinolytics were anaerobic clostridia acting as a flax degumming (“*retting*”) agent (Omelyansky 1904). Most of the known pectinolytic microorganisms grow optimally at acidic and neutral pH, while evidence for pectinolysis at high pH and/or high salt are scarce, although potentially useful for application in the food and textile industry. Producers of alkalitolerant pectinases are known among non salt-tolerant aerobes, such as various bacilli (Hoondal et al. 2002). With respect to specific haloalkaline habitats, such as soda lakes and soda soils, only two cases of pectin degradation have been reported so far, both describing novel members of the phylum *Bacteroidetes* and represented by the genera *Alkaliflexus* and *Natronoflexus* (Zhilina et al. 2004; Sorokin et al. 2011). These organisms are low salt tolerant and multisubstrate-utilizing saccharolytic fermentative alkaliphiles able to hydrolyze pectin among other polymers. However, the question remained, whether pectin degradation is possible in saturated soda brines with salinity above 2 M Na^+^.

In this study, we focused on the microbial degradation of pectin and related polymers in anoxic sediments of hypersaline soda lakes and soda soils. The high salt enrichments resulted in the isolation of six strains of haloalkaliphilic clostridia representing a novel phylogenetic lineage at the family level, whose properties are described below.

## Methods

### Samples

Samples of the top 5 cm sediments from five hypersaline soda lakes and 2 soda solonchak soils were taken in Kulunda Steppe (Altai, Russia) in July 2009. The brine pH varied from 9.95 to 10.5, the total salinity from 100 to 250 g l^−1^ and the soluble carbonate alkalinity from 1.1 to 2.5 M. The soils contained 5–7% (w/w) of soluble salts with an alkalinity of 0.2–0.5 M and a pH of 10.1–10.2 of the water extract. The individual sediment and soil samples were mixed in equal proportions to obtain a single sediment and a single soil sample, which were subsequently used as an inoculum to enrich for haloalkaliphilic pectinolytics.

### Enrichment and cultivation of anaerobic pectinolytic haloalkaliphiles

Enrichment and cultivation of haloalkaliphilic pectinolytics was performed at 28°C on a mineral medium containing sodium carbonate buffer (0.6–3.0 M Na^+^) with pH 10 (stable after sterilization) and 0.5 g l^−1^ of K_2_HPO_4_. After sterilization at 120°C for 30 min, the medium was supplemented with 10 mg l^−1^ of yeast extract, 4 mM NH_4_Cl, 1 mM MgSO_4_, and 1 ml l^−1^ each of acidic trace metal solution and vitamin mix (Pfennig and Lippert 1966). Carbohydrates were added at concentrations 1 g l^−1^ from 10% (w/v) sterile stock solutions (sugars were filter-sterilized, and polymers were autoclaved at 110°C for 30 min at neutral pH). Anaerobic cultivation was performed either in 15 ml Hungate tubes with 10 ml medium, or in 50 ml serum bottles with 40 ml medium with argon in the gas phase. The tubes and bottles were closed with black butyl rubber stoppers and made anoxic by 5 cycles of evacuation–argon flushing with final addition of 1 mM HS^−^ as a reductant. Solid medium was prepared by 1:1 mixing of the complete liquid media and 4% (w/v) washed agar at 50°C. The plates were incubated in closed jars under argon with an oxygen-consuming catalyzer (Oxoid). The pH dependence for growth was examined at Na^+^ content of 0.6 M, using the following filter-sterilized buffering systems: for pH 6–8, 0.1 M HEPES + NaCl/NaHCO_3_; for pH 8.5–11, a mixture of sodium bicarbonate/sodium carbonate containing 0.1 M NaCl. Because the pH was changing during cultivation (mostly at highest starting values), the final values at the end of the exponential growth phase were taken into consideration of the growth pH profiles. To study the influence of salt concentration on growth, sodium carbonate media at pH 10, containing 0.2 and 4.0 M of total Na^+^ were mixed in different proportions. The polymers used were apple pectin (Sigma), polygalacturonic acid (Sigma), pectic and polypectic acids (Baker). Although according to the chemical textbooks, the latter three substances have the same chemical composition, a difference was observed in their microbial utilization, which indicated different properties. Pectin was sterilized as 10% (w/v) suspension at 110°C, while polygalacturonates were first washed several times in distilled water, resuspended at 5% (w/v), solubilized by neutralization with 2 M NaOH and sterilized by filtration. Upon addition to alkaline media, the polymers formed an insoluble amorphous precipitate. Prolonged incubation of the polymers in alkaline media at pH between 10 and 11 and temperature up to 40°C did not result in any detectable spontaneous release of reducing sugars into the medium.

### Analyses

The growth experiments were performed in duplicate. Growth was determined by following an increase in optical density at 600 nm (OD_600_) and the evidences for polymer degradation was observed by gradual disappearance of the precipitate in comparison with the uninoculated controls. In the case of cultures grown on pectin substrates, the solids were removed before the cell density measurements by a brief low-speed centrifugation. The growth rate was measured during the exponential increase of the biomass. Fermentation products were analyzed by HPLC anionic chromatography (BioRad, HPX-87-H column at 60°C, eluent 5 mM H_2_SO_4_ solution at 0.6 ml min^−1^, UV and RI detectors) after neutralization of the supernatant. Reducing sugars were detected by the nitrosalicylate method (Miller 1959) after the removal of solids by high-speed centrifugation. Hydrolytic activity was qualitatively assayed by the agar diffusion method. Essentially, the cultures were grown at pH 10 with polygalacturonate until its complete consumption and the cells were harvested by centrifugation and disrupted by sonication to prepare the cell-free extract. The supernatant was filter-sterilized and 10 times concentrated on Centricon spin filter (Millipore) with pore size of 10 kDa. The fractions were applied into the wells cut in 1% agar containing the pH buffers used for cultivation (see above) with the final Na^+^ concentration 0.6 M and supplied with 1 g/l of polygalacturonate. The plates were incubated at 30°C for 8 h anaerobically inside the gas-tight containers and the zones of hydrolysis were visualized by staining with 0.05% Ruthenium Red (Gang et al. 2010). Phase-contrast microphotographs were obtained with a Zeiss Axioplan Imaging 2 microscope (Göttingen, Germany). Polar lipids for fatty acid composition were extracted from 1 g of wet cell pellet with acidic methanol and the fatty acid methyl esters were analyzed by GC–MS according to Zhilina et al. (1997). For the total polar lipid analysis, the cells were extracted with chloroform–methanol (1:2, v/v) on ice twice and the polar lipid fraction was resolved by two-dimensional TLC (Kieselgel 60, 10 × 10 cm, Merck) using chloroform–methanol–water (60:25:4) in the first direction, followed by chloroform–acetic acid–methanol–water (85:15:12:4) in the second direction. Plates were sprayed with various specific reagents for detection of different phospholipids (Kates 1972). Catalase activity was measured iodimetrically according to Sumner and Dounce (1963).

The isolation of the DNA and determination of the G+C content of the DNA was performed according to Marmur (1961) and Marmur and Doty (1962), respectively. DNA–DNA hybridization was performed by thermal spectrophotometry according to De Ley et al. (1970). For molecular analysis, the DNA was extracted from the cells using alkaline SDS lysis at 60°C and purified with the Wizard Preps Kit (Promega, USA). The nearly complete 16S rRNA gene was obtained using the general bacterial PCR primers 11f and 1492r (Lane 1991). The sequences were aligned with sequences from GenBank using CLUSTAL W and a phylogenetic tree was reconstructed using the neighbour-joining algorithm in the TREECONW program package (van de Peer and de Wachter 1994).

## Results and discussion

### Enrichment and isolation of pectinolytic haloalkaliphiles

**Fig. 1:**
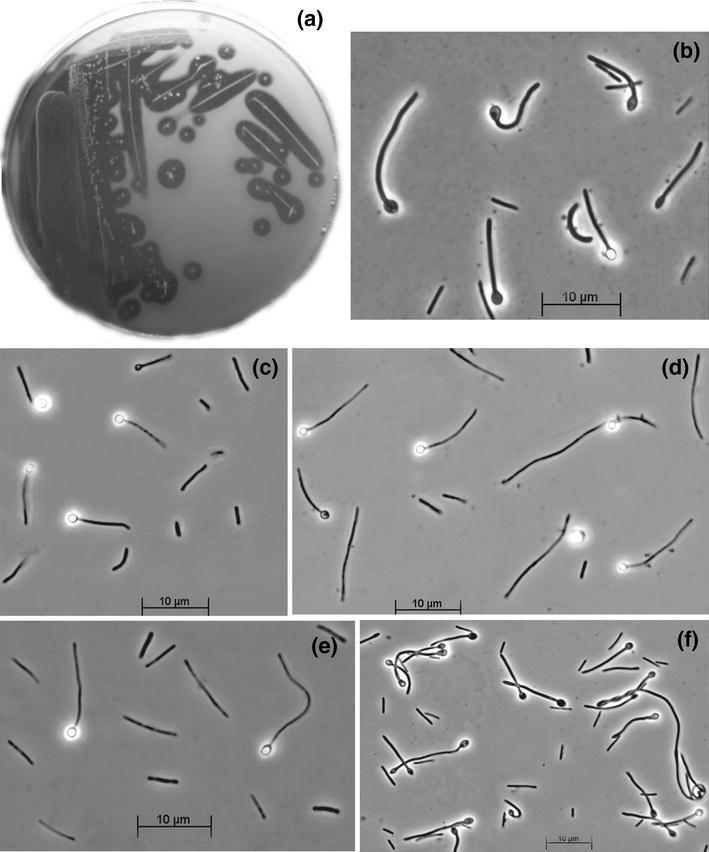
Morphology of haloalkaliphilic pectinolytic isolates grown at pH 10. **a** Formation of clearance zones around colonies of strain AP3 during growth on polygalacturonate. Cell morphology (phase-contrast microphotographs): **b** strain APP2, **c** strain AP3, **d** strain APG1, **e** strain APG2, **f** strain APA1

Anaerobic enrichments at pH 10 with apple pectin and linear polymers of galacturonic acids using soda lake sediments and soda soils as inoculum were positive at salt concentrations up to 4 M Na^+^, indicating a potential for anaerobic pectin degradation up to soda-saturating conditions. However, the growth at 4 M Na^+^ was extremely slow and pure culture isolation failed at these conditions. At salt concentrations between 0.6 and 3 M Na^+^, the enrichments were stable and were dominated by clostridial morphotypes with long rod-shaped cells forming terminal spore. Plating of these enrichments resulted in colony formation with characteristic clearance zone indicating the ability to hydrolyze insoluble pectin polymers (Fig. 1a). As a result, six pure cultures were obtained dominating in six different enrichments (Table 1). All of them were represented by rod-shaped non-motile cells with variable length forming terminal ellipsoid or round endospores (Fig. 1b–f).

**Table 1:** Pectinolytic strains isolated from soda lakes and soda soils in Kulunda Steppe at pH 10

Strain	Source	Enrichment conditions		DSM number	16S rRNA gene accession number
		Total Na^+^ (M)	Polymer substrate		
APG1	Lake sediments	0.6	Polygalacturonate	DSM24177	GQ863486
APG2	Soda soils	0.6	Polygalacturonate		JN801139
APA1	Lake sediments	0.6	Pectic acid		JN801138
APP2^T^	Lake sediments	3.0	Polypectate	DSM24176	GQ863487
AP2	Lake sediments	2.0	Apple pectin		GQ922845
AP3^T^	Soda soils	0.6	Apple pectin	DSM24629	GQ922846

### Identification

**Fig. 2:**
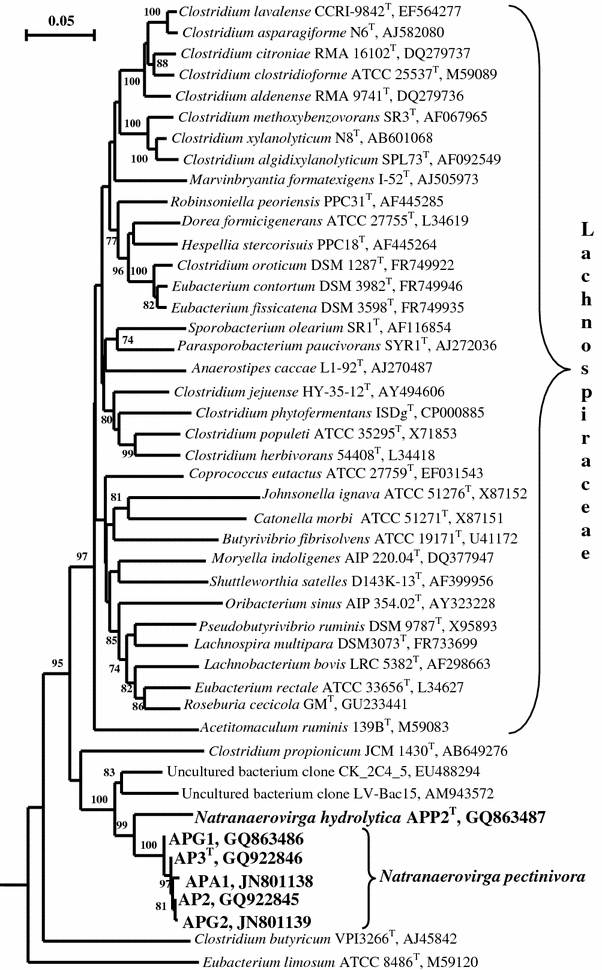
Phylogenetic position of haloalkaliphilic pectinolytic strains within the order *Clostridiales* based on 16S rRNA gene sequence analysis. Tree topography and evolutionary distances are obtained by the neighbor-joining method with Jukes and Cantor distances. The *scale bar* represents 5 nucleotide changes per 100 nucleotides. The *numbers* on the nodes indicate bootstrap values above 70%

Phylogenetic analysis based on 16S rRNA gene sequencing demonstrated that all six isolates belong to the order *Clostridiales* forming a deep lineage (max. 89% sequence similarity to described species) with two subgroups (APP2 and the other 5 strains) with sequence similarity between the subgroups of 94–95%. Preliminary Blast analysis showed that the novel lineage was distant from all ten currently recognized families of the order *Clostridiales* but appeared to be loosely associated with the members of the family *Lachnospiraceae* (Cotta et al. 2009; Rainey 2009) (Fig. 2; Supplementary Fig. 1). Among them were several “representatives” of the genera *Clostridium* and *Eubacterium*, which, on the first glance, is confusing. However, closer inspection clearly showed, that this are misclassified members of the family *Lachnospiraceae*. The association with this family of the novel isolates was also evident from the RDP Classifier, which gave 93% probability for the clustering. On the other hand, the position of the novel group will remain uncertain until more sequences of related organisms appear in the database. It is worth to notice that some of the representatives of this family are typical anaerobic pectinolytics, such as members of the type genus *Lachnospira* (Rainey 2009). Strains APP2^T^ and AP3^T^ were selected as the representative type strains of the two subgroups. The level of DNA–DNA similarity between strain AP3, AP2 and APA1 was from 82 to 90% confirming their belonging to a single genetic species.

Chemotaxonomic comparison showed general similarity between PLFA (polar lipids fatty acids) profiles of the type strains and a closest cultured representative of the family *Lachnospiraceae* (*Robinsoniella peoriensis*) with a domination of 16:0 and 16:1ω7c (Table 2). On the other hand, the profiles of novel isolates differed from typical clostridia by the absence of C14 species. This might be a specific feature of alkaliphilic clostridia. The polar lipid profiles of the type strains were relatively complex and also significantly different from each other. The identified components included phosphatidylglycerol and diphosphatidylglycerol. Furthermore, both strains contained an aminophospholipid, a range of unidentified phospholipids (APP2—8, AP3—5), unidentified glycolipids (APP2—1, AP3—3) and unidentified polar lipids (APP2—1, AP3—2) (Supplementary Fig. 2).

**Table 2:** Comparison of the PLFA profiles of strains AP3 and APP2 and its closest relative (Cotta et al. 2009) and a typical clostridial species (Park et al. 2001)

Fatty acid	AP3	APP2	*Robinsoniella peoriensis*	*Clostridium butyricum*
11:0				1.3
13:1			2.7	
3-OH13:0			**8.2**	
14:0			**11.4**	**9.3**
a15	2.2			3.5
15:0	1.6	2.0		4.9
15:0a				1.8
16:1ω5	0.8	1.3		
16:1ω7a		1.4		
16:1ω7c	**11.5**	**10.7**	**11.9**	
16:1ω7t	0.6			
16:1ω9	2.4	3.7		
16:1ω9a	1.7	**4.8**		
16:0	**46.9**	**57.8**	**32.6**	**31.1**
16:0a		1.1		
a17	0.5			
17:1	0.6			4.3
i17:1			**7.1**	
17:0	1.6			
18:1ω9	0.8		1.4	**6.7**
18:1ω7c	**17.5**	3.7	**16.0**	**12.6**
18:1ω7t	**6.0**			
18:0	1.5		1.3	**6.9**
18:1ω7a	1.5	2.3		
19:0			2.4	2.7
19cyc			2.1	0.8
20:1				1.5

The dominant FA is in bold. Only the values above 0.5% are presented. The alkaliphilic strains were grown up to late logarithmic phase at 30°C, 0.6 M total Na^+^ and pH 10 with galacturonic acid as substrate

### Metabolic characteristics

The novel isolates have a very narrow substrate range and represent extremely specialized obligately anaerobic fermenters able to utilize only pectin (except strain APP2), polygalacturonates and galacturonic acid as growth substrates. In addition, strain APP2 utilized glucuronic acid. None of the following carbohydrates supported growth: d-glucose, d-fructose, galactose, d-mannose, d-maltose, sucrose, dextrose, α, α-trehalose, melibiose, melizitose, l-arabinose, l-sorbose, d-raffinose, d-ramnose, d-glucosamine, *N*-acetyl glucosamine, α-methyl-glycoside, 2-desoxyglucose, d-lactose, d-ribose, d-xylose, m-inositol, m-erythritol, l-arabinite, d-cellobiose, glycogen, starch, dextrine, xylan, laminarin, pullulan, alginate, CMC, and cellulose.

The enzyme complex apparently responsible for the hydrolysis of pectin and its analogs was detected both in the culture supernatant and in the cell fraction by the agar diffusion method, although the activity was obviously higher in the cell-free supernatant (Supplementary Fig. S3). The activity was optimal between the pH 9 and 10.

The main difference between strain APP2 and the other 5 strains was the preference of the latter subgroup for polymers over the monomer galacturonic acid, which was utilized much slower and usually after a prolonged lag phase. Such extreme substrate specialization has been reported for one of the pectinolytic (pectinophilic in author’s interpretation) representatives of the *Clostridiales*, *Lachnospira pectinoshiza* (Cornick et al. 1994). The final products of galacturonic acid fermentation were acetate and formate. H_2_ was detected only in trace amounts and CO_2_ was not possible to detect because of the highly alkaline conditions. No growth was observed in the presence of oxygen in the gas phase and it was stimulated by the addition of reductants. Catalase activity was not detectable.

### Influence of pH and sodium on the growth and activity

**Fig. 3:**
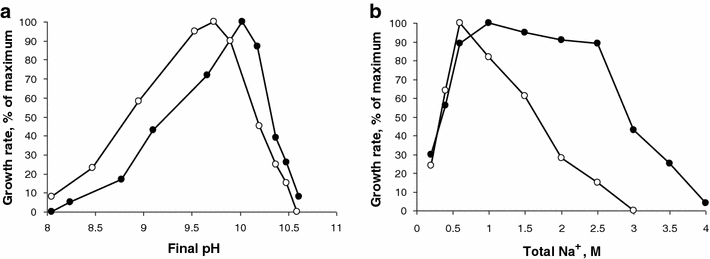
Influence of pH at 0.6 M Na^+^ (**a**) and of sodium carbonate at pH 10 (**b**) on anaerobic growth of strains AP3 (*open circles*) and APP2 (*closed circles*) with galacturonic acid. The experiments were performed in duplicates and the *points* represent the average values with standard deviations below 10%. The initial pH values were the following: 8.0 (final 8.1); 8.5 (final 8.5–8.7); 9.0 (final 9.0–9.18); 9.5 (final 9.53–9.66); 9.75 (final 9.73); 10.0 (final 9.91–10.02); 10.25 (final 10.18–10.20); 10.5 (final 10.37); 10.65 (final 10.48); 10.8 (final 10.59–10.61)

With galacturonic acid as substrate, the strains AP3 and APP2 grew at a pH between 8–8.2 and 10.5–10.6 with an optimum at pH 9.7–10 (Fig. 3a). The growth was chloride independent. In sodium carbonate buffer at pH 10, growth occurred between 0.2 and 2.5 M total Na^+^ with an optimum at 0.4–0.6 M in strains AP3, APA1 and APG1 and between 0.2 and 3.5 M Na^+^ with an optimum at 1.0 M in strain APP2 (Fig. 3b). According to these characteristics, the organisms belong to the high salt-tolerant obligate alkaliphiles.

The overall properties of the anaerobic haloalkaliphilic pectinolytic strains isolated from soda habitats of south-eastern Siberian soda lakes, including large phylogenetic divergence from the closest described relatives, high salt tolerance, obligate alkaliphily and extreme metabolic specialization (summarized in comparative Table 3) suggest accommodation of the six strains into a novel genus *Natranaerovirga* within the order *Clostridiales* with uncertain family affiliation. Five closely related strains (AP2, AP3^T^, APG1, APA1 and APG2) comprise a type species *Natranaerovirga pectinivora*, while a single strain APP2^T^ forms a second species *N. hydrolytica*.

**Table 3:** Phenotypic comparison of strains AP3^T^ and APP2^T^ with the members of the family *Lachnospiraceae*

Characteristic	AP3^T^	APP2^T^	*Robinsoniella peoriensis* ^a^	*Lachnospira pectinoshiza* ^b^
Cell size (µm)	0.25–0.3 × 3–10	0.25–0.4 × 4–10	0.2–0.4 × 4.0–22.0	0.3–0.5 × 2–6
Endospores	+, terminal, round	+, terminal, round	+, subterminal	+, subterminal, round
Motility	–	–	–	+
Major fermentation products	Acetate, formate	Acetate, formate	Acetate, formate, succinate, lactate, ethanol	Acetate, formate
Substrates				
Galacturonic acid	+	+	n.d.	–
Pectin	+	–	n.d.	+
Polygalacturonates	+	+	n.d.	+
Other sugars	–	Glucuronic acid, fructose	Multiple	Fructose, lactose, cellobiose
Maximal growth temperature	43	45	n.d.	45
pH range (optimum)	8.0–10.5 (9.5–9.7)	8.2–10.6 (10)	6.9–9.3 (7.8)	Neutrophile
Salt range (M Na^+^)	0.2–2.5	0.2–3.5	Non-halophilic	Non-halophilic
Polar lipids	PG, DPG, PL, GL, APL	PG, DPG, PL, GL, APL	PG, DPG, GL, APL, APGL, PE	n.d.
Dominant fatty acids in polar lipids	16:0, 16:1ω7c, 18:1ω7c	16:0, 16:1ω7c,	14:0, 16:0, 16:1ω7c, 18:1ω7c	n.d.
G+C content (mol%)	30.7	32.0	48.7	42.0
Habitat	Soda soils	Soda lake sediments	Marine	Marine

*n.d.* not determined, *PG* phosphatidylglycerol, *DPG* diphosphatidylglycerol, *PE* phosphatidylethanolamine, *PL* phospolipids, *APL* aminophospholipids, *GL* glycolipids, *APGL* aminophosphoglycolipids

^a^Cornick et al. (1994)

^b^Cotta et al. (2009)

## Electronic supplementary material

Below is the link to the electronic supplementary material.

Supplementary material 1 (PDF 422 kb)

## Appendix 1

### Description of *Natranoerovirga* gen. nov.

[Natr.anaero’vir.ga; N.L. n. *natron* (arbitrarily derived from the Arabic n. *natrun* or *natron*) soda, sodium carbonate; N.L. pref. *natrono*-, pertaining to soda; Gr. pref. *an*, not; Gr. n. *aer* *aeros*, air; L. fem. n. *virga*, rod; N.L. fem. n. *Natranaerovirga*, an anaerobic rod living in soda].

Gram-positive spore-forming rods with variable length. Obligately anaerobic with fermentative metabolism. Utilize polymers of galacturonic acid as growth substrates. Obligately alkaliphilic and highly salt tolerant. Belong to the order *Clostridiales*. Habitats—soda lakes and soda soils. The type species is *Natranaerovirga pectinivora*.

## Appendix 2

### Description of *Natranaerovirga pectinivora* sp. nov.

(pec.ti.ni’vo.ra N.L. n. *pectinum*, pectin; L. part. adj. *vora*, devouring; N.L. part. adj. *pectinivora*, pectin-devouring).

Cells are nonmotile rods with variable length, 0.35–0.40 × 2–20 µm. Gram-positive, forming ellipsoid to round terminal endospores. Strictly anaerobic fermentative saccharolytic bacteria, utilizing only galacturonic acid and its polymers, such as polygalacturonates and pectin. The final products of galacturonic acid fermentation are acetate and formate. Obligately alkaliphilic with a pH range for growth between 8.0 and 10.5, and an optimum at pH 9.7–10. High salt tolerant with a range from 0.2 to 2.5 M Na^+^ (optimum at 0.4–0.6 M). Mesophilic, with a maximum temperature for growth at 41–43 (type strain 43)°C. Catalase-negative. The polar lipids in type strain consist of phosphatidylglycerol, diphosphatidylglycerol and a range of unidentified polar, phospho-, glyco- and aminophospholipids. The predominant fatty acids in the polar membrane lipids of the type strain include C_16:0_, C_18:1ω7c_ and C_16:1ω7c_. The G+C content of the genomic DNA is 30.3–31.1 (type strain is 30.7) mol% (*T*_m_). The type strain is AP3^T^ (DSM24629^T^=UNIQEM U805^T^). Isolated from soda solonchak soils in south-western Siberia. The GenBank 16S rRNA gene sequence accession number of the type strain is GQ922846.

## Appendix 3

### Description of *Natranaerovirga hydrolytica* sp. nov.

(hyd.ro’ly.ti.ca Gr. n. *hydor,* water; Gr. adj. *lytikos,* dissolving, splitting; N.L. adj. *hydrolytica*, polymer hydrolyzing).

Cells are nonmotile rods with variable length, 0.5–0.6 × 2.5–15 µm. Gram-positive, forming round terminal endospores. Strictly anaerobic fermentative saccharolytic bacterium, utilizing only galacturonic and glucuronic acids, fructose and polygalacturonates. The final products of galacturonic acid fermentation are acetate and formate. Obligately alkaliphilic with a pH range for growth between 8.2 and 10.6, and an optimum at pH 10. High salt tolerant with a range from 0.2 to 3.5 M Na^+^ (optimum at 1 M). Mesophilic, with a maximum temperature for growth at 45° C. Catalase-negative. The polar lipids in type strain consist of phosphatidylglycerol, diphosphatidylglycerol and a range of unidentified polar, phospho-, glyco- and aminophospholipids. The predominant fatty acids in the polar membrane lipids of the type strain include C_16:0_, and C_16:1ω7c_. The G+C content of the genomic DNA is 32.0 mol% (*T*_m_). The type strain is APP2^T^ (DSM24176^T^=UNIQEM U806^T^). Isolated from soda lake sediments in south-western Siberia. The GenBank 16S rRNA gene sequence accession number of the type strain is GQ863487.
