# Supplementary material for: Anaerobic utilization of pectinous substrates at extremely haloalkaline conditions by Natranaerovirga pectinivora gen. nov., sp. nov., and Natranaerovirga hydrolytica sp. nov., isolated from hypersaline soda lakes
Source: Extremophiles. 2012 Feb 1;16(2):307–15. doi: 10.1007/s00792-012-0431-6 (PMC3325495; doi:10.1007/s00792-012-0431-6)
Supplement: Supplementary file 1 — Supplementary material 1 (PDF 422 kb) [file 792_2012_431_MOESM1_ESM.pdf]

Supplementary Fig.S1. Extended 16S rRNA gene-based phylogenetic tree constructed by the maximum likelihood algorithm showing affiliation of the genus "Natranaerovirga" within the order *Clostridiales* [the family set up of the order *Clostridiales* is according to : Ludwig W, Schleifer K-H, Whitman WB (2006) Revised road map to the phylum *Firmicutes*. In: Bergey's Manual of Systematic Bacteriology, Second Edition, Volume 3: The *Firmicutes*, pp. 1-15]

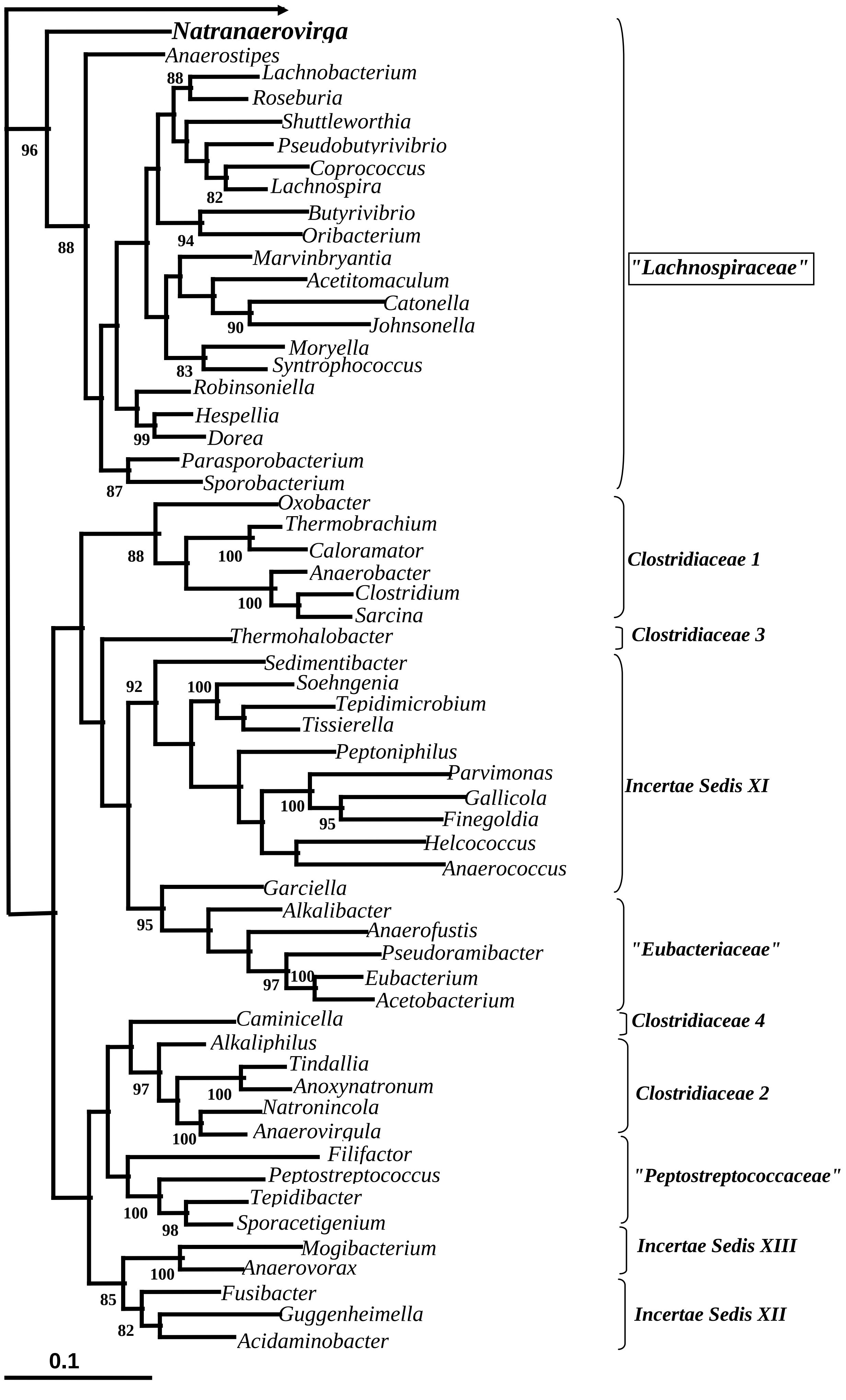

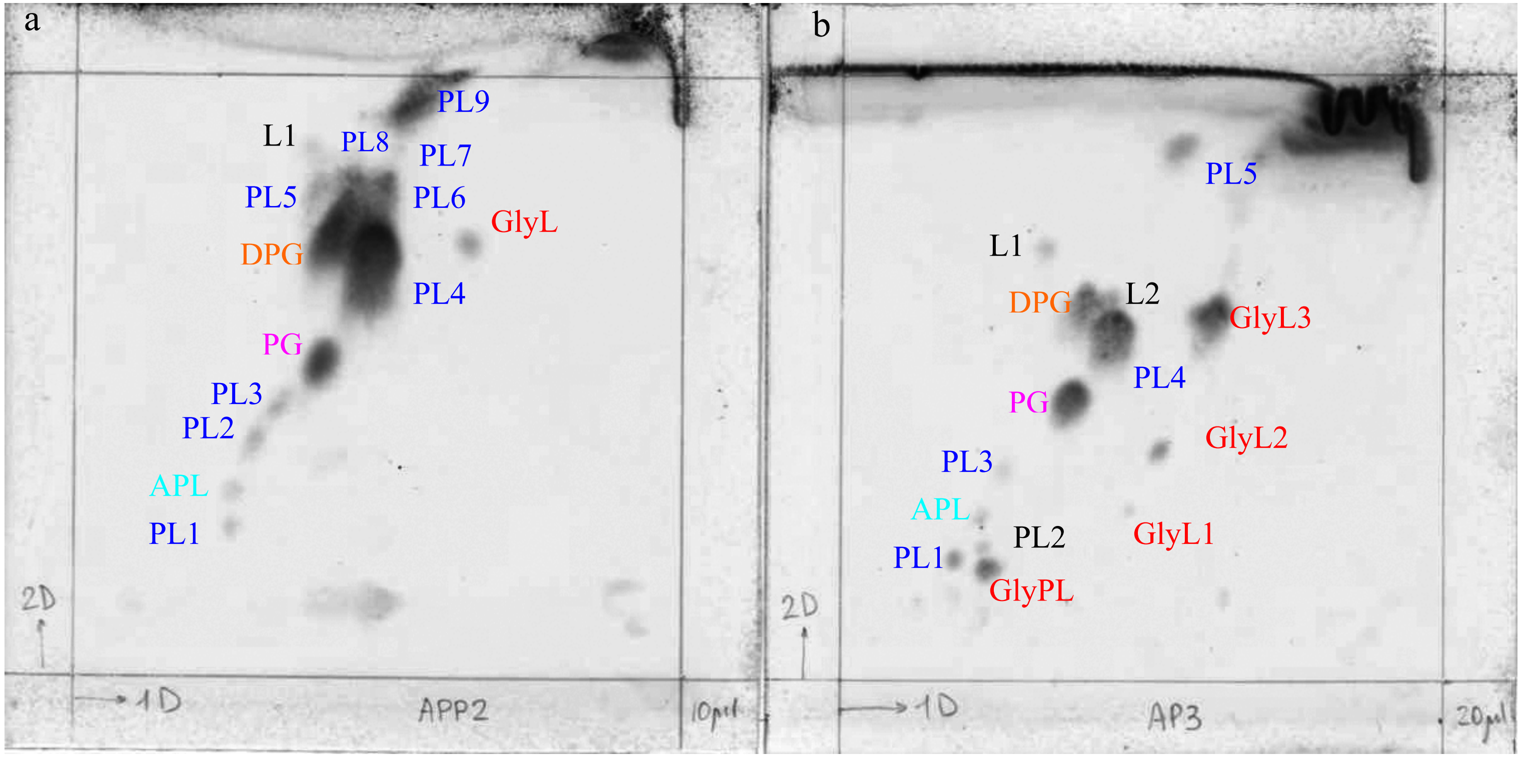

Supplementary Fig. S2. Two-dimensional TLC of polar lipids of strains APP2^{T} (a) and AP3^{T} (b). Chloroform/methanol/water (65:25:4) was used in the first direction, followed by chloroform/acetic acid/methanol/water (80:15:12:4) in the second direction.

For detection of total lipids, the plate was sprayed with 5% ethanolic molybdatophosphoric acid. Molybdenum blue (Sigma), ninhydrin and α-naphthol-sulphuric acid reagent were used for the detection of phospholipids, aminolipids and glycolipids, respectively.

Abbreviations: PG, phosphatidylglycerol; DPG, diphosphatidylglycerol; GlyPL, unknown glycophospholipid; GlyL1-3, unknown glycolipids; APL, unknown aminophospholipid; L1-2, unknown polar lipids; PL1-9, unknown phospholipids.

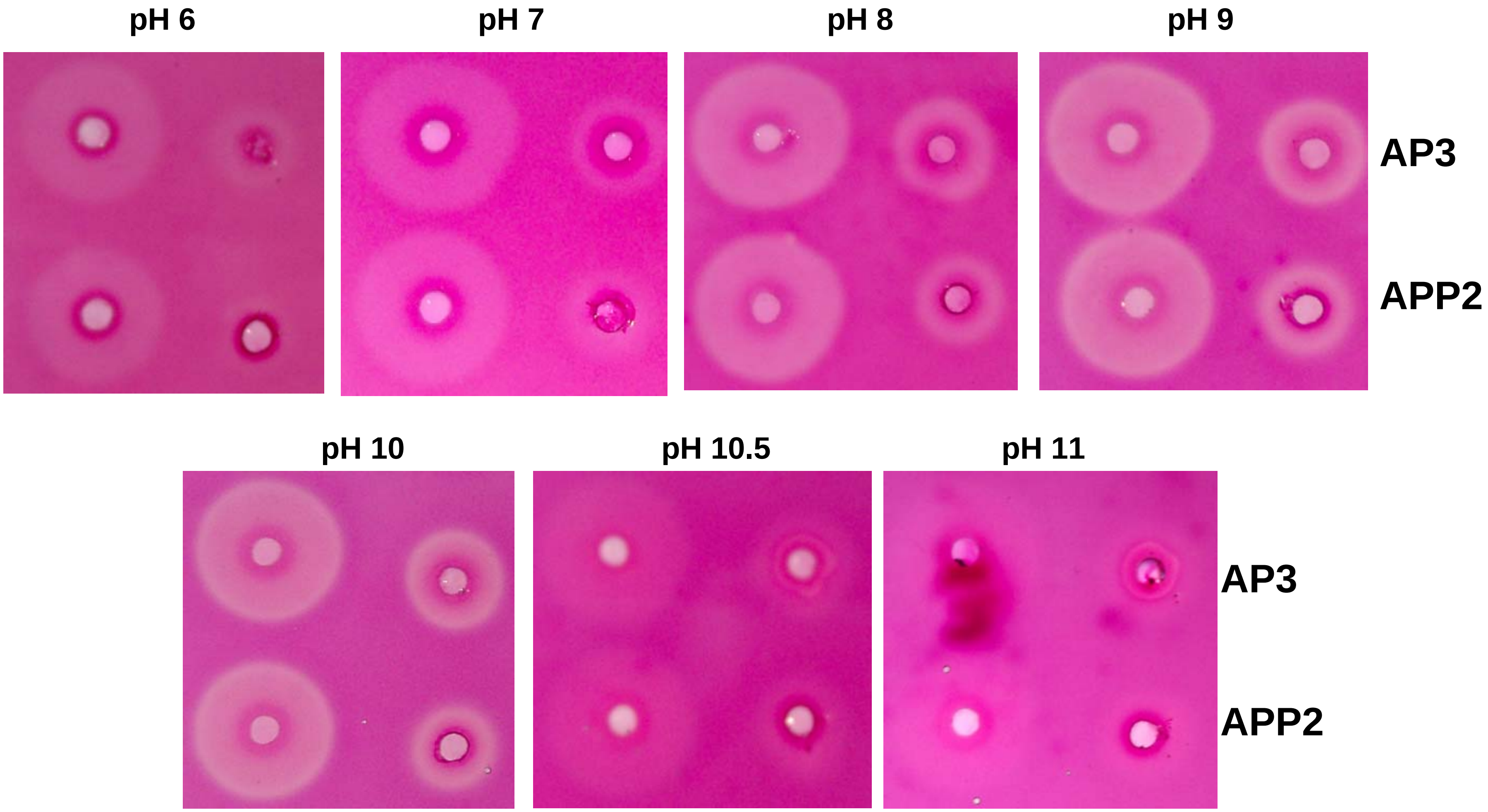

Supplementary Fig. S3. Pectinlyase activity (with polygalacturonate) in cell-free fractions of haloalkaliphilic anaerobes at different pH demonstrated by the agar diffusion method. Incubation temperature is 30°C, incubation time is 8 h. Stain is Ruthenium Red according to : [Gang et al., J. Microbiol. Biotechnol. (2010), 20(4), 670–677]. Left spots: culture supernatant concentrated 10 times with the 10 kDa Centricon spin filters; AP3=1.1 μg protein, APP2=1.2 μg protein. Right spots: cell-free extract: AP3= 12 μg protein, APP2= 10 μg protein.
